# Design and Synthesis of Cofacially-Arrayed Polyfluorene Wires for Electron and Energy Transfer Studies

**DOI:** 10.3390/molecules28093717

**Published:** 2023-04-25

**Authors:** Rajendra Rathore, Sameh H. Abdelwahed

**Affiliations:** 1 Department of Chemistry, Marquette University, Milwaukee, WI 53233, USA; 2 Department of Chemistry, Prairie View A&M University, Prairie View, TX 77446, USA

**Keywords:** charge transfer, energy transfer, semiconducting, π-stacking, polyfluorene, oligofluorene, cation radicals, electrochemistry, cyclic voltammetry, ultraviolet-visible-near-infrared (UV-vis-NIR) spectroscopy

## Abstract

A study of cofacially arrayed π-systems is of particular importance for the design of functional materials for efficient long-range intra-chain charge transfer through the bulk semiconducting materials in the layers of photovoltaic devices. The effect of π-stacking between a pair of aromatic rings has been mainly studied in the form of cyclophanes, where aromatic rings are forced into a sandwich-like geometry, which extensively deforms the aromatic rings from planarity. The synthetic difficulties associated with the preparation of cyclophane-like structures has prevented the synthesis of many examples of their multi-layered analogues. Moreover, the few available multi-layered cyclophanes are not readily amenable to the structural modification required for the construction of D–spacer–A triads needed to explore mechanisms of electron and energy transfer. In this review, we recount how a detailed experimental and computational analysis of 1,3-diarylalkanes led to the design of a new class of cofacially arrayed polyfluorenes that retain their π-stacked structure. Thus, efficient synthetic strategies have been established for the ready preparation of monodisperse polyfluorenes with up to six π-stacked fluorenes, which afford ready access to D–spacer–A triads by linking donor and acceptor groups to the polyfluorene spacers via single methylenes. Detailed ^1^H NMR spectroscopy, X-ray crystallography, electrochemistry, and He(I) photoelectron spectroscopy of F2–F6 have confirmed the rigid cofacial stacking of multiple fluorenes in F2–F6, despite the presence of rotatable C–C bonds. These polyfluorenes (F2–F6) form stable cation radicals in which a single hole is delocalized amongst the stacked fluorenes, as judged by the presence of intense charge-resonance transition in their optical spectra. Interestingly, these studies also discern that delocalization of a single cationic charge could occur over multiple fluorene rings in F2–F6, while the exciton is likely localized only onto two fluorenes in F2–F6. Facile synthesis of the D–spacer–A triads allowed us to demonstrate that efficient triplet energy transfer can occur through π-stacked polyfluorenes; the mechanism of energy transfer crosses over from tunneling to hopping with increasing number of fluorenes in the polyfluorene spacer. We suggest that the development of rigidly held π-stacked polyfluorenes, described herein, with well-defined redox and optoelectronic properties provides an ideal scaffold for the study of electron and energy transfer in D-spacer-A triads, where the **Fn** spacers serve as models for cofacially stacked π-systems.

## 1. Introduction

**Figure 1:**
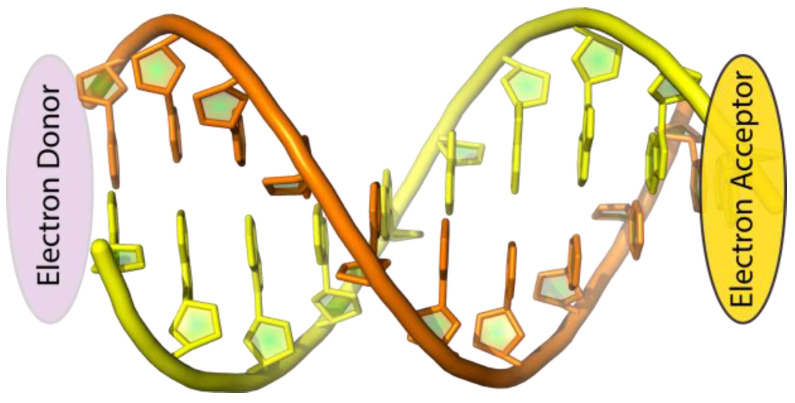
Donor–DNA–acceptor assemblies for electron-transfer studies through DNA wires.

Through-space interactions between cofacially stacked aromatic rings are at the origin of many phenomena of photovoltaics and biological chemistry. For example, the role of cofacially stacked π-systems has recently played out prominently in studies of long-range charge transfer in DNA through stacked aromatic bases, as well as in the exploration of DNA’s use in futuristic molecular electronic devices (Figure 1) [1]. After much controversy surrounding the efficiency of long-range charge transfer through stacked bases in DNA, the current consensus, reached largely through the work of researchers at Northwestern University [2], is that DNA can transport charge via a hopping mechanism through π-stacked bases [3,4,5,6,7].

**Figure 2:**
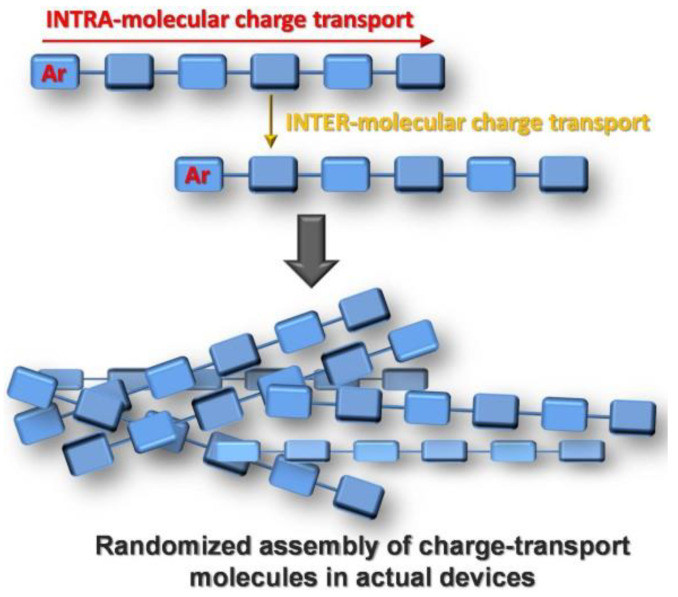
A cartoon diagram showing the *intra-chain* versus *inter-chain* charge transfer in bulk semiconductors.

While the suggested application of DNA in modern photovoltaic devices is yet to be realized, usage of cofacially stacked organic π-systems could instead be potentially important in photovoltaic devices, i.e., organic solar cells and light emitting diodes [1]. For example, since the discovery of conducting polyacetylenes [8], a variety of π-conjugated organic polymers, e.g., poly-*p*-phenylenes, polythiophenes, polyphenylenevinylenes, and polyfluorenes [9,10,11,12,13,14], have been employed in charge-transport layers of the photovoltaic devices. It is expected that the efficiency of charge transport in the conducting layers of these devices is governed by not only the intra-chain charge transport through the backbone of π-conjugated polymers but also via inter-chain charge transport through the bulk material, where the assembly of π-conjugated molecules is expected to be randomized (Figure 2) [15,16,17,18,19]. Therefore, development of the next generation of conductive organic materials for photovoltaic applications requires careful study of the model compounds to develop a fundamental understanding of structure–function relationships for the inter-chain charge transport through cofacially stacked aromatic π-systems.

## 2. Cyclophanes as Models for the Study of π-Stacking

**Figure 3:**
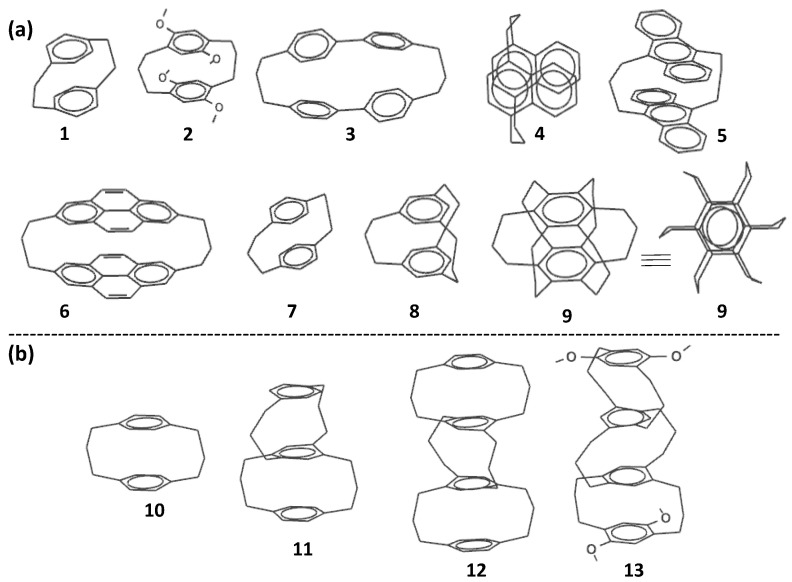
(**a**) Structures of representative cyclophanes derived from the benzenoid and polynuclear hydrocarbons, and (**b**) examples of multi-decker cyclophanes.

The π-stacking or through-space interactions between a pair of cofacially stacked aromatic rings has been studied primarily in the cyclophanes, molecules where two π-systems are forced into a sandwich-like geometry that generally imparts extensive deformation of the cofacial aromatic moieties (Figure 3) [20]. The chemistry of cyclophanes was first initiated by Cram [21], who showed that the two benzene rings in paracyclophane **1** lie at a distance shorter than the van der Waals contact. Expectedly, such a close cofacial approach of a pair of benzene rings in **1** alters its electronic and photophysical properties owing to the efficient electronic coupling [21].

Since this initial discovery, a variety of cyclophanes containing polynuclear aromatic hydrocarbons (**2–6**) with varying bridge sizes have been synthesized (Figure 3) [20]. A recent successful multi-step synthesis of a six-bridge “superphane” **9** (Figure 3) provides testament to the continually evolving power of modern synthetic methodologies; however, electronic coupling between aromatic rings in **9** is dramatically modulated via the sigma framework [22], rendering it a non-ideal model for the evaluation of π-stacking.

## 3. Multi-Decker Cyclophanes

**Figure 4:**
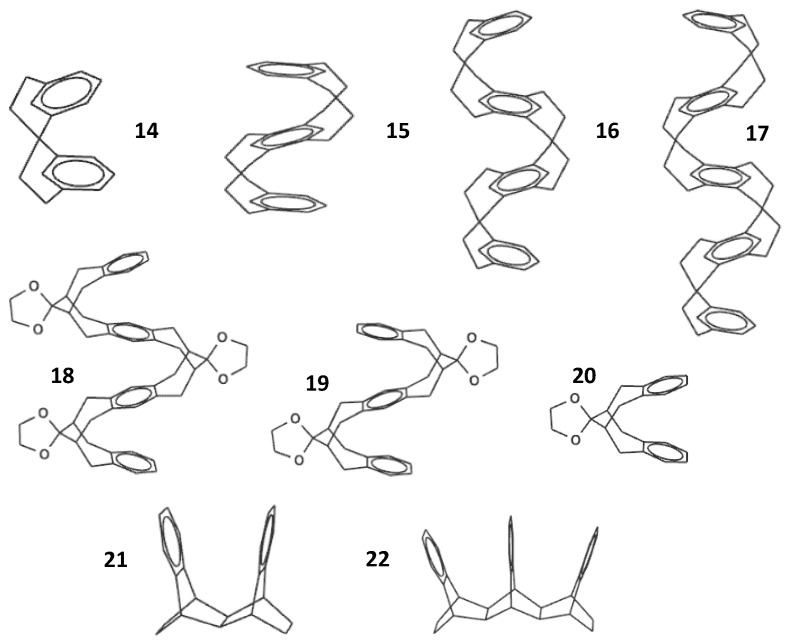
Structures of representative multi-decker cyclophanes.

Due the synthetic difficulties, examples of cyclophanes with multiple layers are scarce (e.g., Figure 3b) [23]. Unfortunately, the few reported systems (**11–13**) exist as a mixture of stereoisomers, and the benzene rings are significantly distorted from planarity [23]. Examples of multi-layered cyclophanes in which the benzene rings are largely planar have also been prepared via multi-step syntheses (Figure 4); however, these studies have been generally limited to cyclic voltammetry and donor–acceptor complexation with tetracyanoethylene as an electron acceptor [24,25,26].

A hallmark of electronic coupling in these systems is the lowering of the redox potential by efficient stabilization of the cationic charge. While the aromatic rings in the cyclophane-like molecules **14–22** in Figure 4 do not suffer distortions from planarity, their interplanar angles are far from the sandwich-like geometries found in cyclophanes **1–13** (Figure 3). It is therefore of interest that the reversible oxidation potentials of a series of cyclophane-like bichromophoric molecules **28–30** (Table 1) all show a similar thermodynamic stabilization of the cationic charge by ~350 ± 30 mV, despite varied interplanar angles (0–30°).

**Figure 5:**
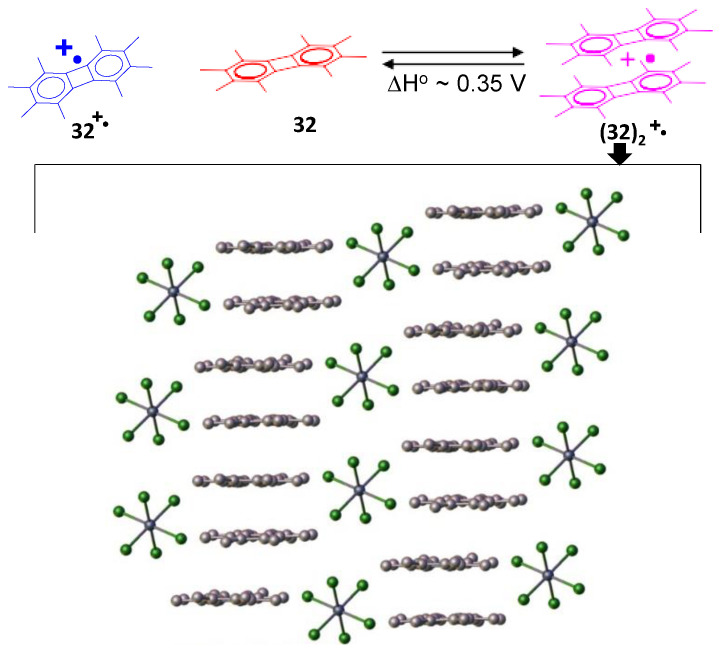
Formation of dimeric (**32**)_2_^+•^ and its crystallographic packing diagram [16].

In comparison, an intermolecularly formed dimer cation radical of octamethylbiphenylene (**32**), displaying a perfect sandwich-like geometry and free of distortions of aromatic rings, also showed an enthalpic stabilization of a single cationic charge by ~350 mV (Figure 5) [16]. It is noted that the dimerization constants of formation of intermolecular (**32**)_2_^+•^ (*K*~300 M^−1^ or Δ*G*o~−150 mV) as well as other dimeric cation radicals [16,27] are relatively low because of the significant entropic penalty for the formation of the intermolecular dimer (i.e., for (**32**)_2_^+•^, *T*Δ*S*^o^ = ~200 mV at 22 °C) [16], a penalty that is absent in the rigid cyclophane-like donors **28–31**.

Electrochemical evaluation of various cofacially arrayed bichromophoric systems (Table 1) against the intermolecularly formed dimer cation radical of **32** (Figure 5) clearly suggests that a close but not complete sandwich-like geometry between interacting π-systems is necessary for effective electronic coupling. Consistent with this expectation, the drastically reduced overlap between the aryl moieties in ethanoanthracene **31**, due to the large interplanar angle of 120°, leads to reduced stabilization of the cationic charge in its cation radical (i.e., ~250 mV) compared with the corresponding model electron donor **27** (Table 1) [28].

## 4. Rationale for the Design of a New Class of Cofacially π-Stacked Polyfluorenes

**Figure 6:**
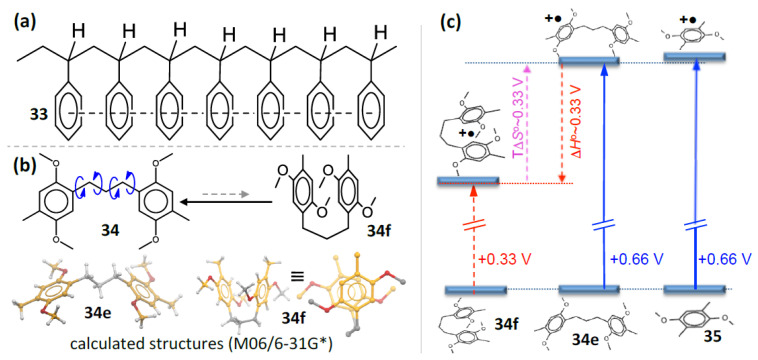
(**a**) Structure of stereo-regular polystyrene. (**b**) The calculated [M06/6-31G* + PCM(CH_2_Cl_2_)] structures of conformers **34e** and **34f**. (**c**) Comparison of electrochemical potentials of **34** and model **35**. It was assumed that *E*_ox_ of **34f** is the same as cyclophane **28** in Table 1.

From these studies, it is clear that the design and synthesis of new π-stacked molecular arrays without the necessity for perfect sandwich-like arrangement are needed for the study of wire-like materials. This insight led us to explore structures that could be accessed via simpler and more versatile synthetic routes. The description below highlights the evolution of design principles involved in the development of cofacially arrayed π-stacked polyfluorenes with the aid of insights gleaned from 1,3-diarylalkane-type structures. It had been speculated [29] that phenyl groups in stereo-regular polystyrenes (**33**) could acquire a cofacial π-stacked arrangement (Figure 6a). Unfortunately, experimental/computational studies of various model 1,3-diarylalkanes (Figure 6b) clearly showed that folding of extended **34e** to cyclophane-like **34f** is unfavorable [30,31,32]. For example, the (observed) lack of folding of **34e^+•^** into appreciable amounts of **34f^+•^**, together with the similar oxidation potentials of **34** and its model compound **35**, suggests that the anticipated enthalpic gain (~350 mV) is mostly negated by the entropic penalty expended in organizing a structure containing four C-C single bond rotors (Figure 6b).

**Figure 7:**
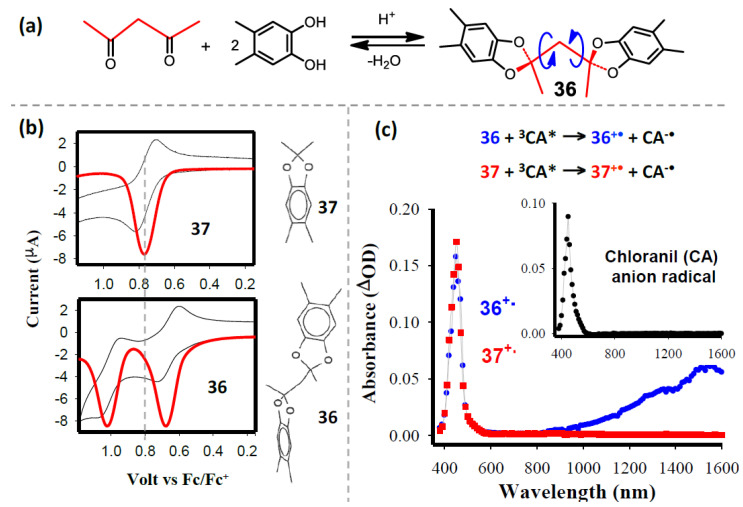
Synthesis (**a**), CVs of **36** (*E*_ox1_ = 0.68 V, *E*_ox2_ = 1.05 V) and **37** (*E*_ox1_ = 0.78 V) in CH_2_Cl_2_ at *v* = 200 mV s^−1^ at 22 °C (**b**), and transient absorption spectra of **36^+•^** (blue) and **37^+•^** (red) generated by laser flash photolysis using triplet chloranil as an oxidant (**c**). Inset: The absorption spectrum of chloranil**^–•^** generated using DABCO as the electron donor whose cation radical absorbs at <350 nm.

**Scheme 1:**
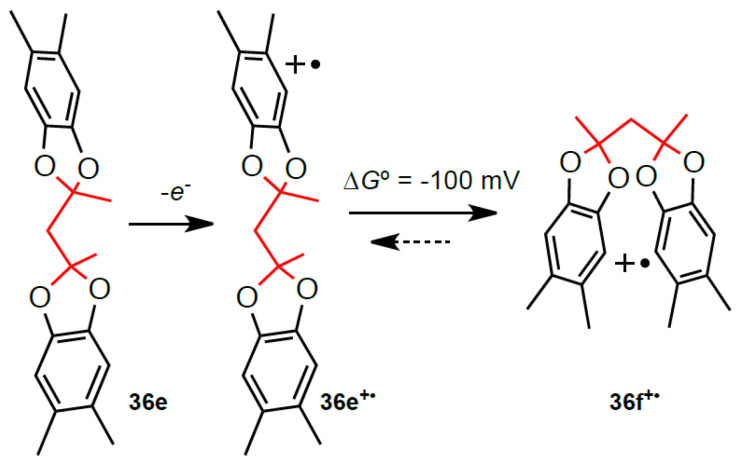
Electron transfe-induced folding of **36e** into **36f^+•^**.

**Figure 8:**
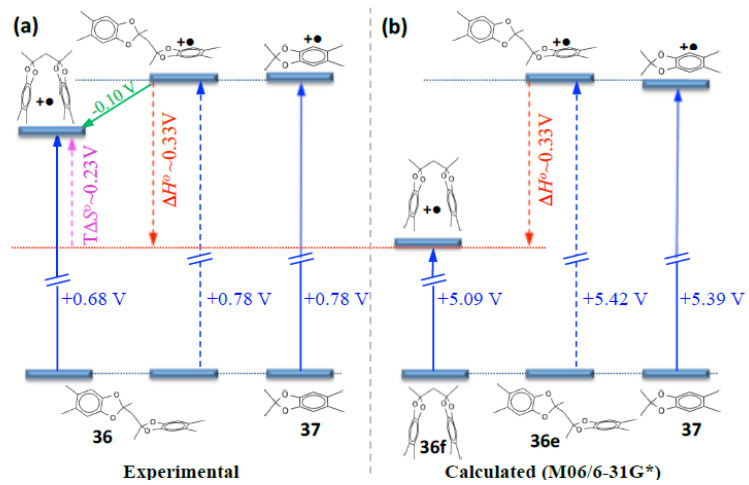
Comparison of the experimental (**a**) and calculated [M06/6-31G* + PCM(CH_2_Cl_2_)] (**b**) oxidation potentials of **36** and model **37**. The oxidation potential of **36e** was assumed to be same as for model **37**.

In order to stabilize a folded cyclophane-like structure, a reduction in the number of C-C bond rotors was required. Thus, we prepared 1,3-diarylalkane **36**, which contains only two C-C single bond rotors (Figure 7a) [33]. Interestingly, the lowering of the first oxidation potential of **36** (~100 mV) compared with that of the model **37** (Figure 7b) suggests that upon 1-electron oxidation, the extended **36e^+•^** must transform instantaneously into a cyclophane-like **36f^+•^**, presumably due to the reduced entropic penalty (Scheme 1) [33]. A comparison of the energetics of the **36e^+•^** → **36f^+•^** transformation by experiment and DFT calculations (Figure 8) clearly demonstrate that the entropic penalty for its folding (TΔ*S*° = 230 mV) is significantly reduced due to the reduced number of C-C bond rotors in **36** in comparison with **34** (Figure 6c).

The absorption spectrum of **36^+•^** shows a characteristic intervalence (charge-resonance) transition [27] at λ_max_ = ~1600 nm, while the spectrum of model **37+•** lacks any absorption in this region (see Figure 7c), thus confirming the **36e^+•^** → **36f^+•^** transformation in Scheme 1 [33].

**Figure 9:**
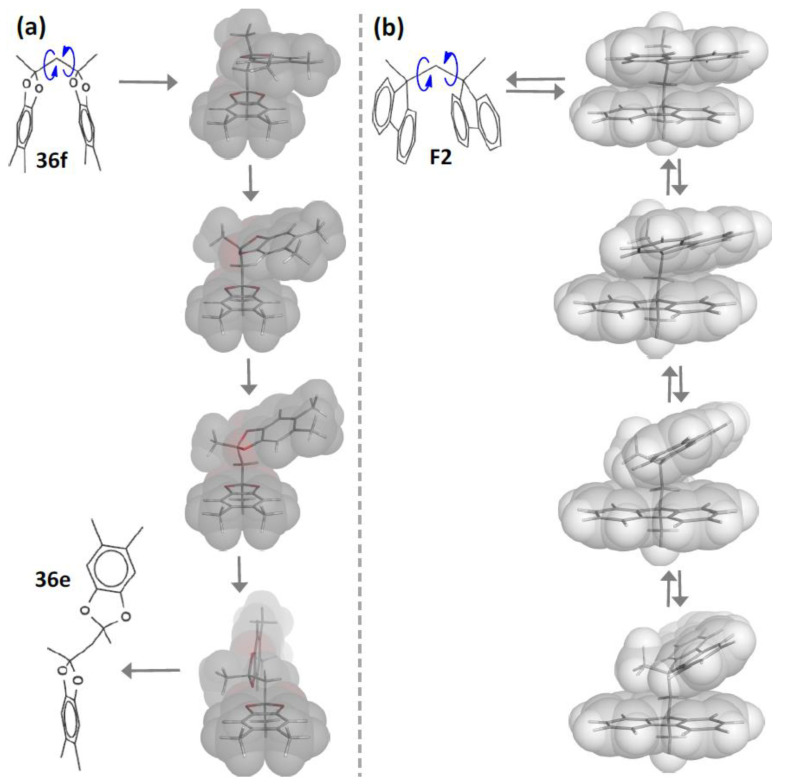
Conformational analysis of (**a**) **36** and (**b**) **F2**, [M06/6-31G* + PCM(CH_2_Cl_2_)].

We then carefully scrutinized the intermediate (transient) structures formed during the **36f** → **36e** transformation by DFT calculations. Figure 9a compiles a series of intermediate structures formed by rotation around one of the two equivalent C-C bond rotors, which shows that the rotation around the C-C bond in **36f** is not significantly impeded by steric crowding of the aromatic rings. Moreover, the slightly lower energy (~2 kcal mol^−1^) of **36e** in comparison with **36f** indicates that it will exist largely in an extended conformer in the neutral state [16,34].

This analysis suggested that increasing the size of the aromatic ‘plate’ in **36** may lead to a 1,3-diarylalkane derivative in which the rotation around the C-C bond will be sterically impeded. Indeed, a conformational analysis of such a derivative, i.e., the difluorene **F2** in Figure 9b, showed that the large size of the fluorene rings and their peri-hydrogens in **F2** pose severe steric crowding for transformation into an extended conformer. Indeed, DFT calculations predict that the extended conformer of neutral **F2** is ~10 kcal mol^−1^ higher in energy than the more stable cofacial conformer.

We therefore undertook synthesis of the cofacially arrayed difluorene **F2** and its higher homologues (**Fn**).

## 5. Development of Synthetic Strategies for the Preparation of π-Stacked Polyfluorene Oligomers

**Scheme 2:**
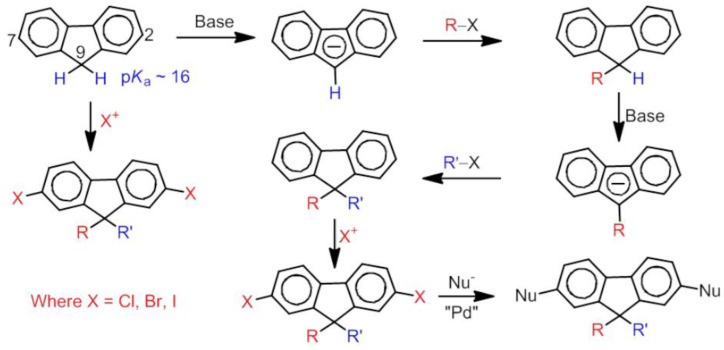
Different functionalization sites in fluorene.

Fluorene is readily available and contains a planar biphenyl unit which can be easily functionalized at its active methylene carbon 9 by nucleophilic substitution, as well as at the activated aromatic carbons 2 and 7 by either electrophilic aromatic substitution or palladium-catalyzed coupling reactions (i.e., Scheme 2).

**Scheme 3:**
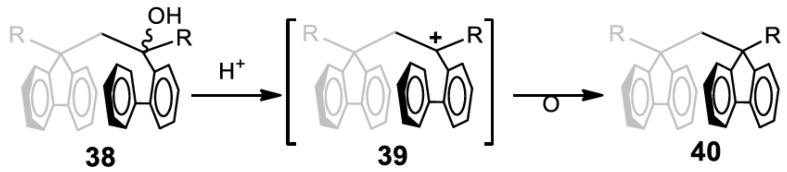
Construction of fluorene ring system by intramolecular Friedel-Crafts cyclization.

It was first thought that the synthesis of **F2** (or its higher homologue **F3**) could be accomplished by use of the intramolecular Friedel-Crafts reaction of the carbocationic intermediate **39**, generated from an alcohol **38** (Scheme 3).

**Scheme 4:**
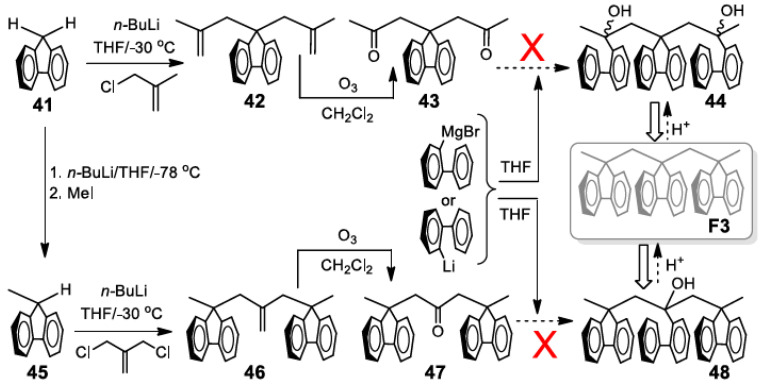
Attempted syntheses of **F3** by Friedel–Crafts cyclization.

Unfortunately, the two conceived Friedel-Crafts precursors to **F3**, i.e., **44** and **48**, from easily synthesized **43** or **47** (Scheme 4), could not be prepared due to the failure of the reactions of either **43** or **47** with 2-biphenylmagnesium bromide or 2-biphenyllithium, most likely due to steric hindrance.

**Scheme 5:**
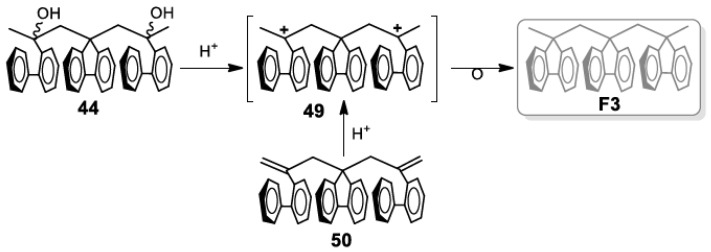
Alternative Friedel–Crafts precursor for **F3**.

The failed synthesis of *bis*-alcohol **44**—a precursor to the dicationic intermediate **49**—led us to explore if it can be accessed by protonation of a corresponding *bis*-alkene derivative **50** (Scheme 5).

**Scheme 6:**
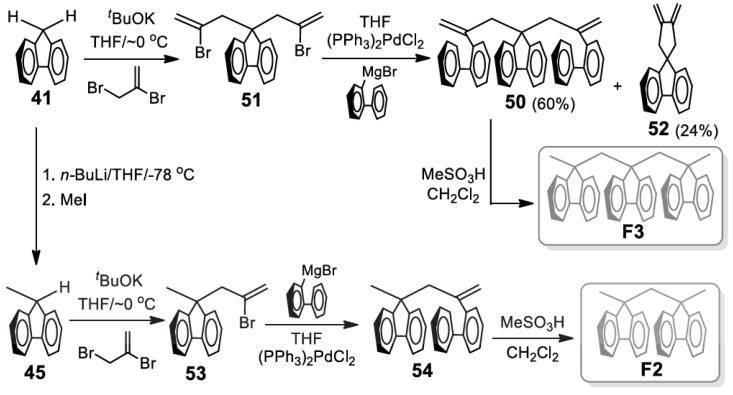
Successful syntheses of **F2** and **F3** via a reliable three-step strategy.

The desired *bis*-alkene **50** was easily synthesized in two steps (Scheme 6); indeed, it underwent a facile acid-catalyzed intramolecular Friedel–Crafts cyclization to produce **F3** in quantitative yield (Scheme 6) [35]. This three-step strategy for the preparation of **F3** from parent fluorene was also applicable to synthesis of **F2** from 9-methylfluorene in ~85% overall yield (Scheme 6).

**Scheme 7:**
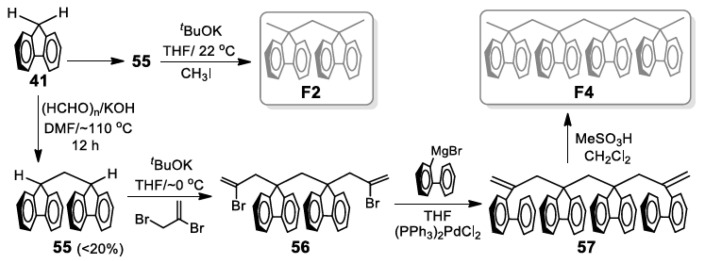
Preparation of **F4** from **55** using a three-step strategy.

The *di*-fluorene **F2** can also be prepared by methylation of the difluorenemethane **55** (Scheme 7). Unfortunately, **55** was obtained in low yield (>20%) after tedious purification of a complex mixture from a reaction of fluorene with paraformaldehyde in the presence of *^t^*BuOK (potassium tert-butoxide) in DMF (Scheme 7) [36]. Notwithstanding the poor yield of **55**, its availability permitted the preparation of the *tetra*-fluorene **F4** with ~70% yield from **55** using the three-step strategy outlined in Scheme 6, i.e., alkylation, Kumada coupling, and Friedel-Crafts cyclization (Scheme 7).

**Scheme 8:**
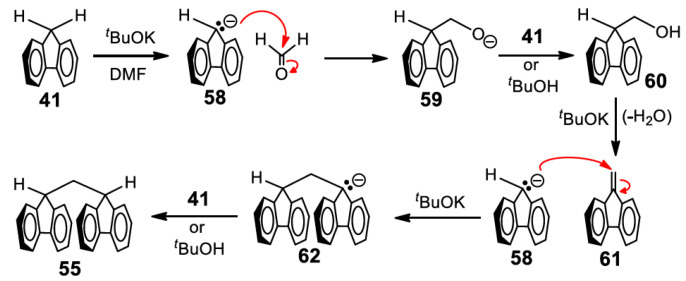
Proposed mechanism for the formation of **55** from **60** and **58**.

A simple mechanistic analysis of the reported preparation of **55** [36] suggested that an initial aldol-type condensation between a fluoranyl anion (**58**) and formaldehyde should produce 9-fluorenomethanol **60** as the initial product followed by formal elimination of water to produce 9-methylenefluorene **61**. A subsequent Michael-type addition of **58** to **61** can produce **55** after protonation, i.e., Scheme 8. Unfortunately, all the reactions depicted in Scheme 8 are expected to be reversible, and therefore, an optimization of the reaction conditions may allow access to **55** and its higher homologues with good yields.

**Scheme 9:**
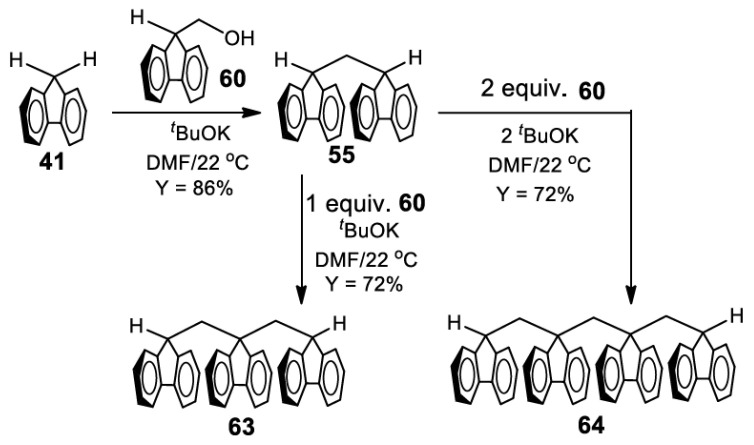
Preparation of **55** and higher homologues.

Surprisingly, a reaction of equimolar (commercially available) fluorenomethanol **60** and fluorene in the presence of *^t^*BuOK as a base in DMF at 22 °C for ~2 min yielded pure **55** after a simple pouring of the reaction mixture into water, filtration, and recrystallization from CH_2_Cl_2_/C_2_H_5_OH, CH_2_Cl_2_/C_2_H_5_OH with >86% yield (Scheme 9) [37,38]. Moreover, the higher homologues **63** and **64** can be similarly prepared from a reaction of **55** with one and two equivalents of **60**, respectively (Scheme 9) [37,38].

**Scheme 10:**
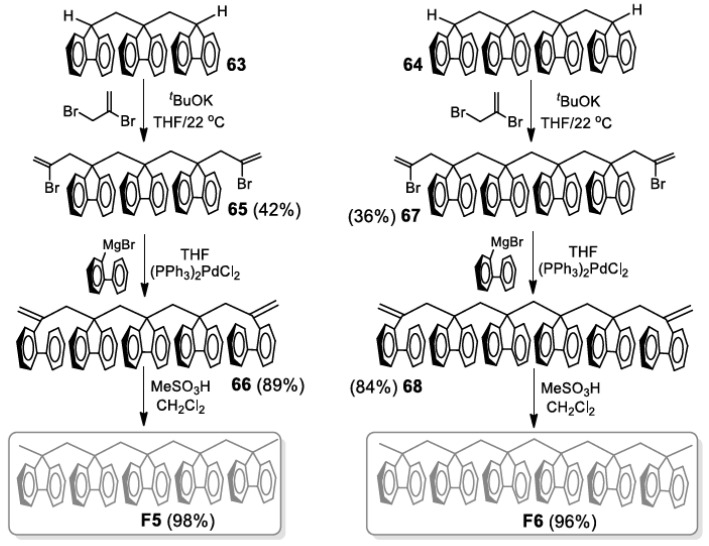
Preparation of **F5** and **F6**.

The availability of **63** and **64** then allowed easy access to **F5** and **F6** using the robust three-step strategy outlined above (Scheme 10). It is noted that somewhat low overall yields of **F5** and **F6** in Scheme 10 arise largely due to partial de-polymerization of **63** and **64** during alkylation. Fortunately, a combination of column chromatography and crystallizations easily produce pure **65** and **67** for further high-yielding transformations to **F5** and **F6** (Scheme 10) [39,40].

**Scheme 11:**
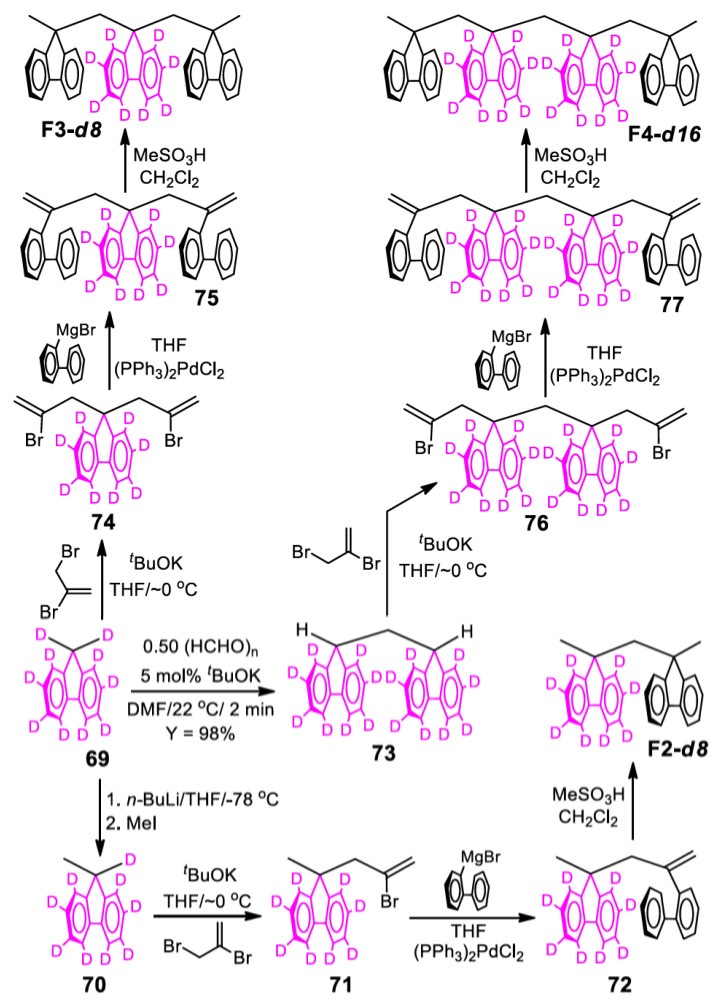
Preparation of selectively deuterated polyfluorenes.

The versatility of the synthetic strategies for the preparation of **F1–F6** (Scheme 6, Scheme 7, Scheme 8, Scheme 9 and Scheme 10) was further delineated by preparation of the selectively deuterated polyfluorenes (e.g., Scheme 11) [39] needed for EPR spectroscopic studies as well as ^1^H NMR spectral assignments (vide infra).

## 6. Demonstration of the Cofaciality of the Fluorene Rings in F1–F6 by ^1^H NMR Spectroscopy

The ^1^H NMR spectroscopy results provide clear evidence of cofacial stacking of fluorenes in **F2–F6** in solution at temperatures from +30 °C to −70 °C.

**Figure 10:**
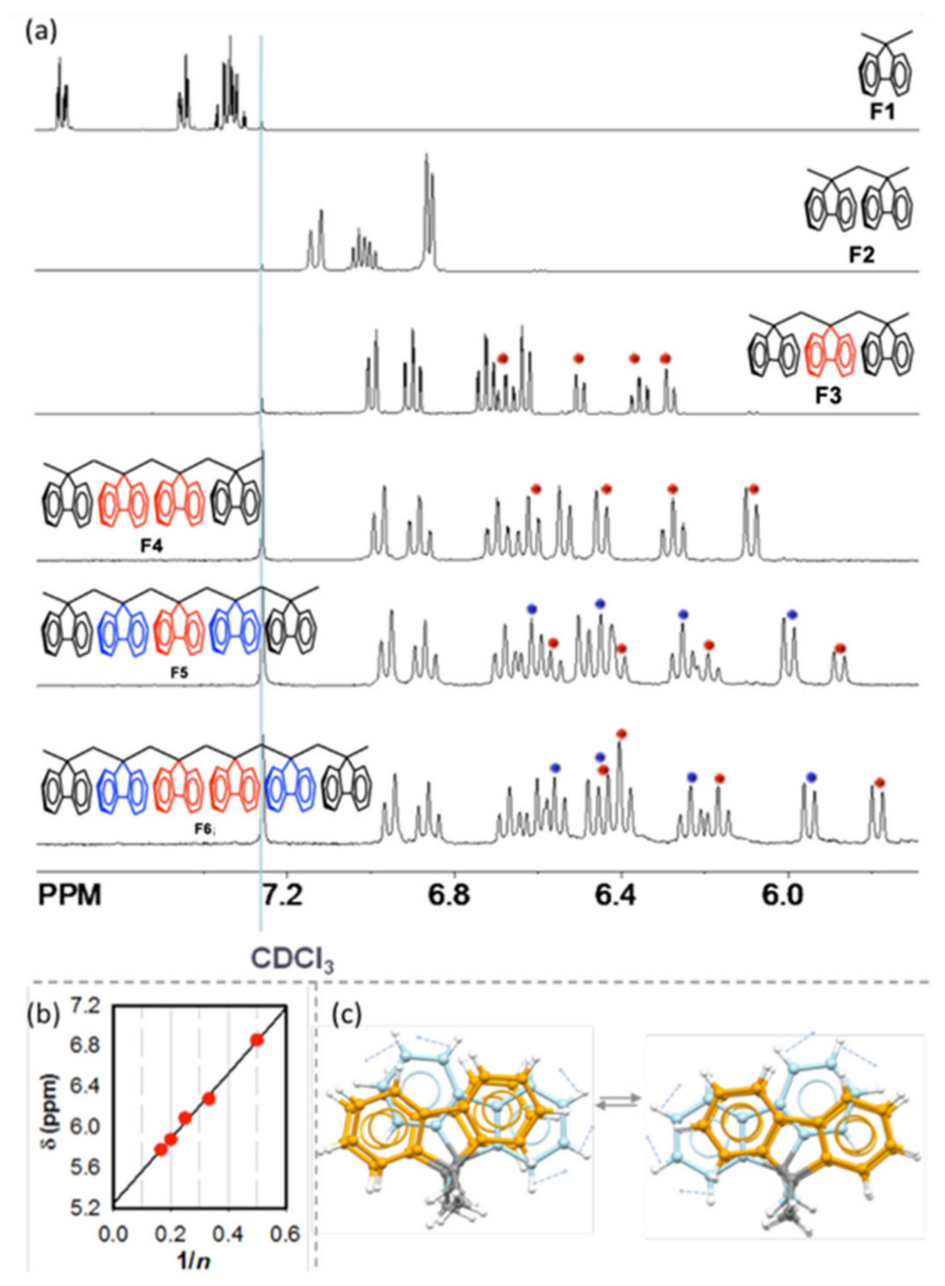
(**a**) Partial ^1^H NMR spectra of **F1–F6** in CDCl_3_ at 22 °C, the cofacial stacked fluorene units were colored based on the anisotropic deshielding (**b**) showing 1/*n* dependence of the chemical shift of H_4_ in **F2–F6** and (**c**) the dynamic displacement of the central fluorene in **F3** from ideal sandwich-like geometry.

**Figure 11:**
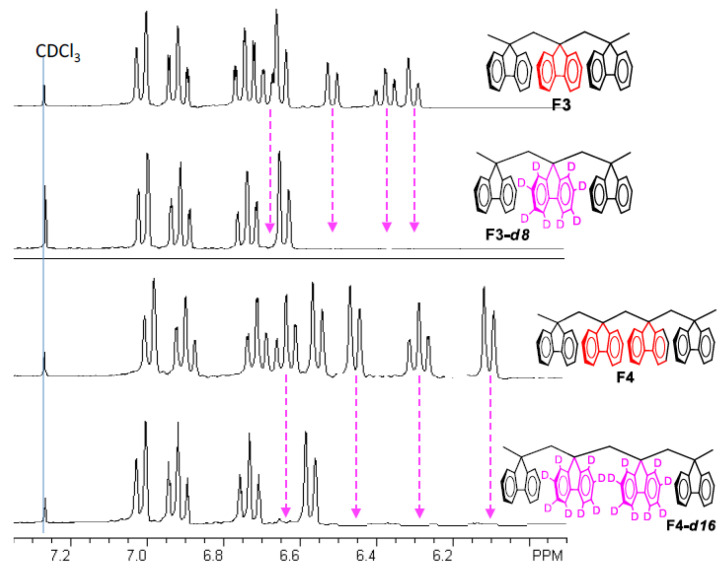
Comparison of ^1^H NMR spectra of **F3-*d8*** and **F4-*d16*** with those of **F3** and **F4**. A further analysis showed that the extent of deshielding of the protons on the central fluorene ring/s in **F2–F6** increases with an increasing number of adjacent fluorenes (Figure 10b). This systematic variation of the chemical shifts due to anisotropic deshielding by adjacent stacked fluorenes also confirms that all the fluorene rings in **F3–F6** are fully π-stacked [35].

For example, a comparison of the ^1^H NMR spectra of **F1–F6** (Figure 10) shows that the fluorene protons in **F2–F6** are shifted up-field compared with the chemical shifts of the corresponding protons in **F1**, which is due to anisotropic deshielding. The assignment of all protons in **F1–F6** was confirmed by 2D NMR spectroscopy as well as by comparison of the NMR spectra of selectively deuterated analogues (e.g., Figure 11) [35,39].

This deshielding of the protons in **F2–F6** arises not due to the perfect sandwich structure but to slightly displaced sandwiches (Figure 10c) which must rapidly interconvert in solution, as the protons on the two benzenoid rings of all fluorenes in **F2–F6** are equivalent on the NMR time scale [35].

## 7. X-ray Crystallography of the π-Stacked Polyfluorenes

**Figure 12:**
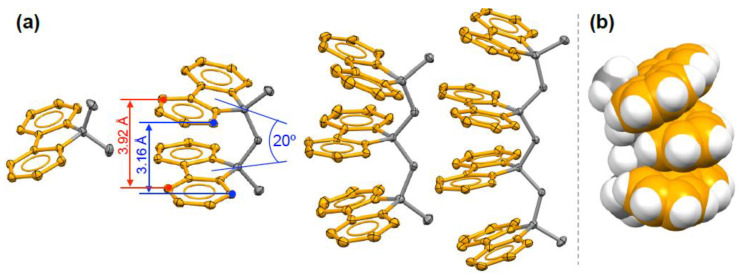
Comparison of the X-ray structures of **F1–F4** (**a**) and space-filling representation of **F3** (**b**).

The molecular structures of **F2–F4**, as determined by X-ray crystallography, showed in each case a close cofacial juxtaposition of the fluorene moieties, with interplanar angles of 18–24° and contacts amongst many of the fluorene carbons that were as close as 2.71–3.18 Å (Figure 12a) [35]. Moreover, a typical space-filling representation of **F3** (Figure 12b) illustrates the close van der Waals contact amongst the cofacially juxtaposed fluorene rings in polyfluorenes. As expected, based on the NMR studies (Figure 10), the solid-state structures of **Fn** show substantial displacement of the fluorene moieties from the ideal sandwich-like geometries, and the alternating direction of this displacement produced achiral structures (Figure 12). Repeated attempts to use chiral additives to crystallize the chiral structures of **F3–F6**, in which the fluorene moieties are systematically displaced on one side, have thus far been unsuccessful.

## 8. Electronic and Emission Spectroscopy of Polyfluorenes

**Figure 13:**
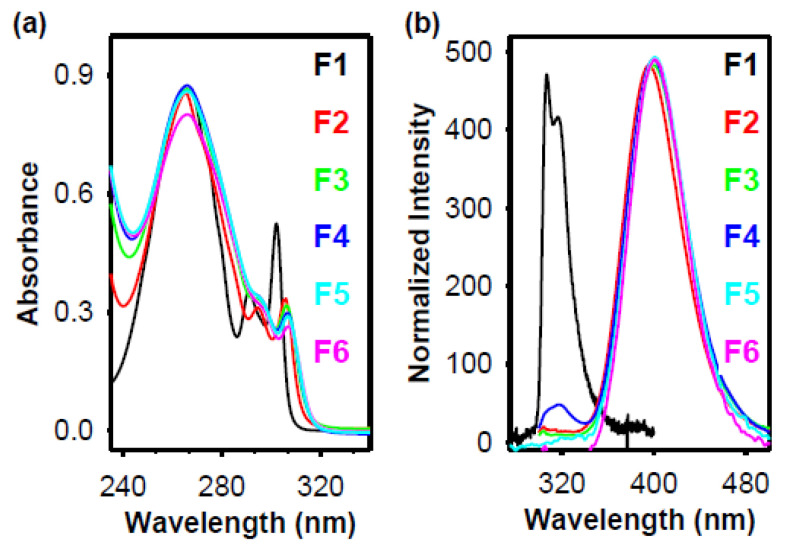
Normalized absorption spectra of (**a**) **F1–F6** with ε_265_ = 17,000; 27,300; 36,000; 45,400; 52,000; and 61,000 M^−1^ cm^−1^; respectively, and emission spectra (**b**) in CH_2_Cl_2_. Note that the relative emission intensity of **F1** was ~10 times higher than for **F2–F6**.

Despite the close van der Waals contacts amongst the fluorene moieties in **F2–F6**, their electronic absorption spectra were rather invariant and were similar to that of monomeric **F1** (Figure 13a) [28]. However, the fluorescence emission spectra of **F2–F6** were strikingly different compared with the spectrum of **F1**. The emission spectrum of **F1** showed a characteristic structured band (307 nm), whereas the remarkably similar emission spectra of **F2–F6** showed a Gaussian band at 395 ± 3 nm (Figure 13b) [35,40].

**Figure 14:**
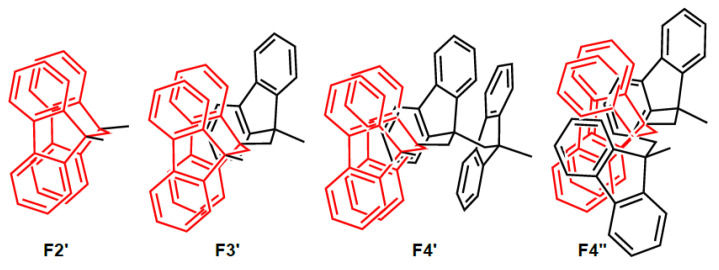
Representative structures showing the localization of the excimeric exciton onto two perfectly sandwiched fluorenes in **F2–F4**. Note that multiple excimeric structures are possible for **F4–F6,** as exemplified by **F4′** and **F4”**.

The striking constancy in the position of the excimer emission band for **F2–F6** suggests that the exciton must reside over only two fluorene moieties [41,42]. The energetic penalty for excimer formation, which requires an ideal sandwich-like arrangement of a pair of interacting units [43], must be easily overcome by the energetic gain due to the excimeric stabilization in **F2**. However, in larger **Fn,** the delocalization of the exciton beyond two units would require energetically demanding rearrangement of three or more fluorene units in ideal sandwich-like geometries. In **F3–F6**, one can expect an ensemble of multiple excimeric structures of similar energy where the exciton is localized only onto two fluorene moieties (e.g., Figure 14).

## 9. Electrochemistry/Photoelectron Spectroscopy of Polyfluorenes

**Figure 15:**
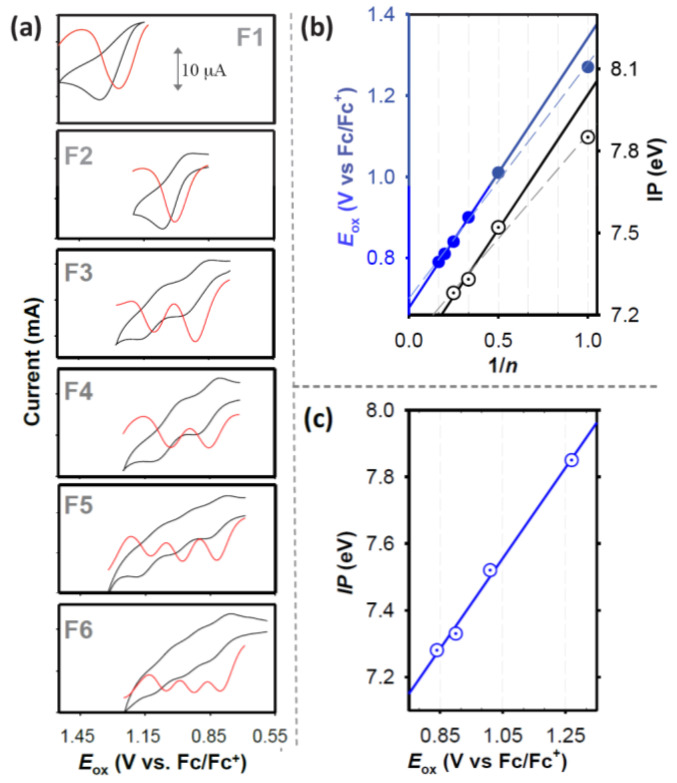
(**a**) Cyclic (black) and square-wave (red) voltammograms of 2 mM **F1–F6** in CH_2_Cl_2_ containing 0.1 M (*n*Bu)_4_NPF_6_ at *v* = 200 mV/s. (**b**) A plot of the IP (black axis) and *E*_ox1_ (blue axis) values versus 1/*n*. Note that dashed lines include **F1** in correlations. (**c**) A plot of IP versus *E*_ox1_ values for **F1–F4**.

Besides **F1**, polyfluorenes **F2–F6** undergo reversible electrochemical oxidation under ambient conditions (Figure 15a) [35,40]. The first oxidation potentials (*E*_ox1_) of **Fn** decrease in order from **F1** to **F6** (i.e., ~1.27, 1.01 0.90, 0.84, 0.81, and 0.79 V vs. Fc/Fc+, respectively), and they show that a single cationic charge is stabilized by ~0.5 V by delocalization over six cofacially stacked fluorenes in **F6** compared with model **F1**.

The He(I) photoelectron spectra of **F1–F4** provided vertical ionization potentials, which decrease in order from **F1** to **F4** (7.85, 7.52, 7.33, and 7.28 eV, respectively), and both *E*_ox1_ values and vertical IPs showed a familiar 1/*n* relationship, with strikingly similar slopes (Figure 15b). Moreover, a Koopmans-like linear relationship (Figure 15c) [44] between thermodynamic oxidation potentials, measured on a submillisecond time scale, and vertical ionization potentials, measured on a subfemtosecond time scale, suggests that stacked **Fns** do not suffer from any conformational reorganization upon one-electron detachment [35,44,45].

Thus, the linear 1/*n* dependence of vertical and adiabatic ionization energies (Figure 15b) and anisotropic chemical shifts (in the ^1^H NMR spectra, Figure 10b) of **F2–F6,** together with X-ray crystallography (Figure 12), demonstrate that this new class of polyfluorenes, designed based on the 1,3-diarylalkane framework, retain their cofacial arrangement in the gas phase, solution, and solid state.

## 10. Generation and Spectroscopy of Polyfluorene Cation Radicals

**Figure 16:**
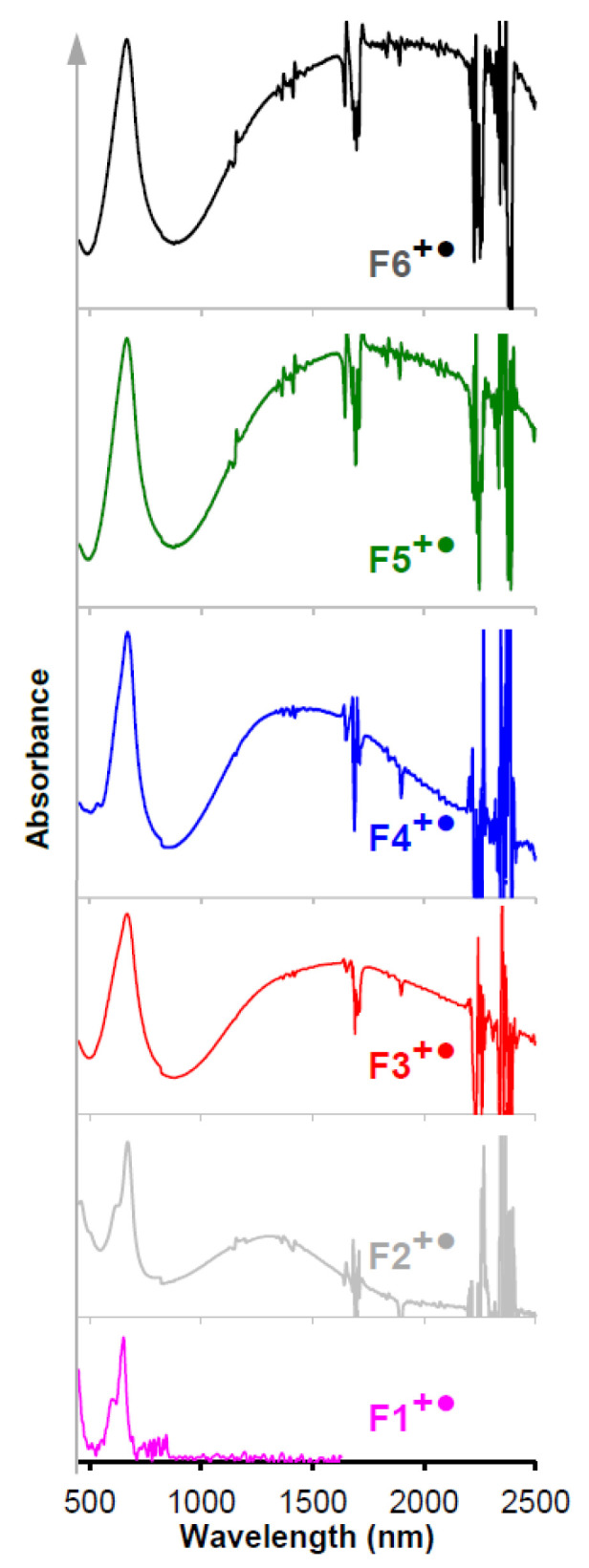
Optical spectra of **F1**^+•^**-F6**^+•^ generated using NO^+^SbCl_6_^−^ in CH_2_Cl_2_ at 22 °C. Note that **F1**^+•^ was generated via laser flash photolysis.

The cofacially stacked **F2–F6** form stable cation radicals when exposed to oxidants such as NO^+^SbCl_6_^−^ [46,47], Et_3_O^+^ SbCl_6_^−^ [48], and SbCl_5_ [49] in CH_2_Cl_2_. The **F1** cation radical, which is highly unstable, was generated by laser flash photolysis using photoexcited triplet chloranil as the oxidant [33,35]. The absorption spectra of **F2**^+•^**-F6**^+•^ in CH_2_Cl_2_ (22 °C) showed characteristic intervalence transitions in the NIR region (Figure 16), which is absent in **F1**^+•^. The intervalence transitions intensify and undergo a red shift with an increasing number of fluorene units, thus confirming increased hole delocalization in the π-stacked fluorene array in **Fn** [50].

This observation of hole delocalization over multiple fluorenes in **Fn** should be contrasted with excimer formation, which is limited to only two fluorene units owing to the requirement for an ideal sandwich-like geometry [43]. Significantly, X-ray crystallography has shown that perfect sandwich-like geometry is not a necessity for the stabilization of the cationic charge in a number of dimeric cation radicals [16]. These facts together suggest that the nature of bonding in excimers and cation radicals is different, possibly due to the different spin multiplicities, and a careful computational study together with X-ray crystallography of **Fn**^+•^ will be needed to address this question.

## 11. Generation of Polyfluorene Anion Radicals and Study of Charge Delocalization by EPR Spectroscopy

**Figure 17:**
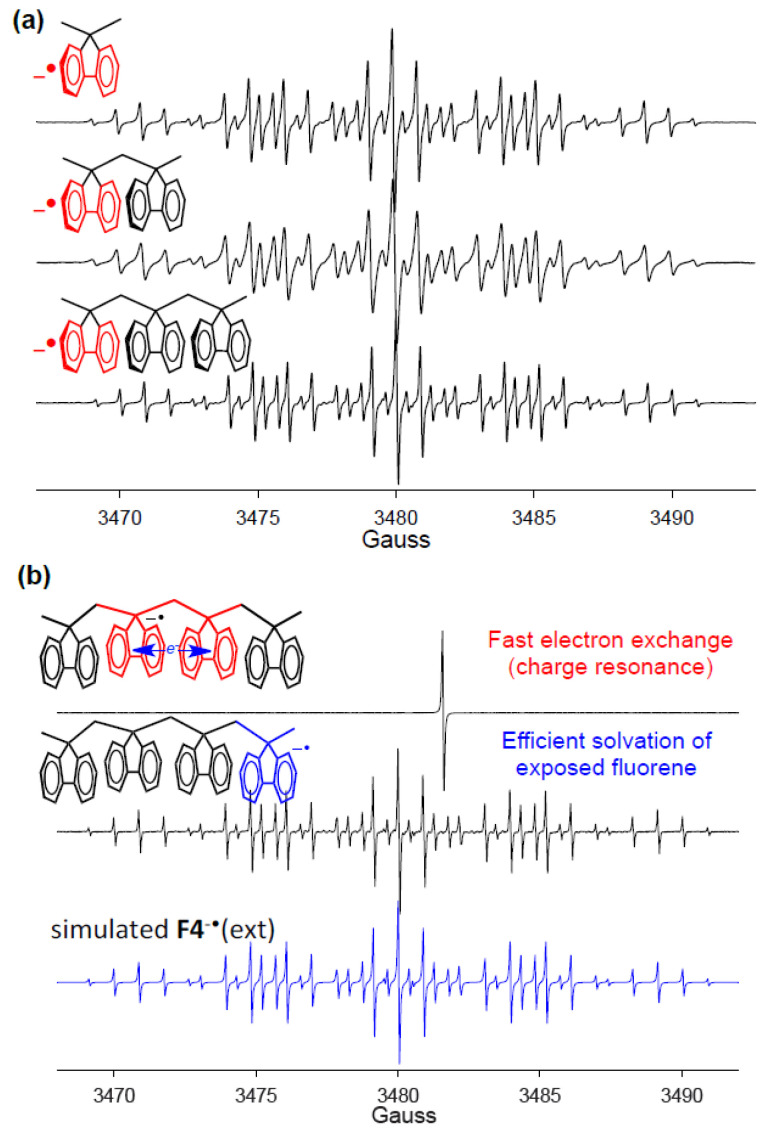
(**a**) The representative EPR spectra of **Fn**-• in HMPA at 22 °C. (**b**) The EPR spectrum of **F4**^−•^(int) obtained immediately after mixing **F4** with potassium (top), the spectrum of the same solution recorded after 1 h (middle), and its simulated spectrum (lower) [39].

To access the anion radicals, we carried out one-electron reduction of **F2–F6** with potassium in HMPA, which was examined using EPR spectroscopy [39,51]. In all cases, the thermodynamically preferred state was formed, where the electron is localized exclusively on one external fluorene (Figure 17a), as judged by the similarity of the EPR spectra with that of **F1**. Interestingly, the initially formed (i.e., kinetically controlled) **F4^−•^**–**F6^−•^** do allow rapid exchange of the electron amongst the internal fluorenes, as discerned by the appearance of a single broad line in their time-dependent EPR spectra (e.g., Figure 17b). These initially formed anion radicals eventually transform into anion radicals where the electron is localized onto a single external fluorene owing to the stringent solvation and ion-pairing requirements for the stabilization of the anionic charge.

This observation was further substantiated by an experiment with partially deuterated **F4**, where the two internal fluorenes were perdeuterated. Here, the transformation of the kinetic to thermodynamically stabilized anion radical occurred at least an order of magnitude faster than in perprotiated **F4** due to the fact that deuterated hydrocarbons stabilize the unpaired electron less effectively [39].

## 12. Donor–Spacer–Acceptor Triads with Polyfluorenes as Spacers and Demonstration of the Triplet Energy Transfer

**Figure 18:**
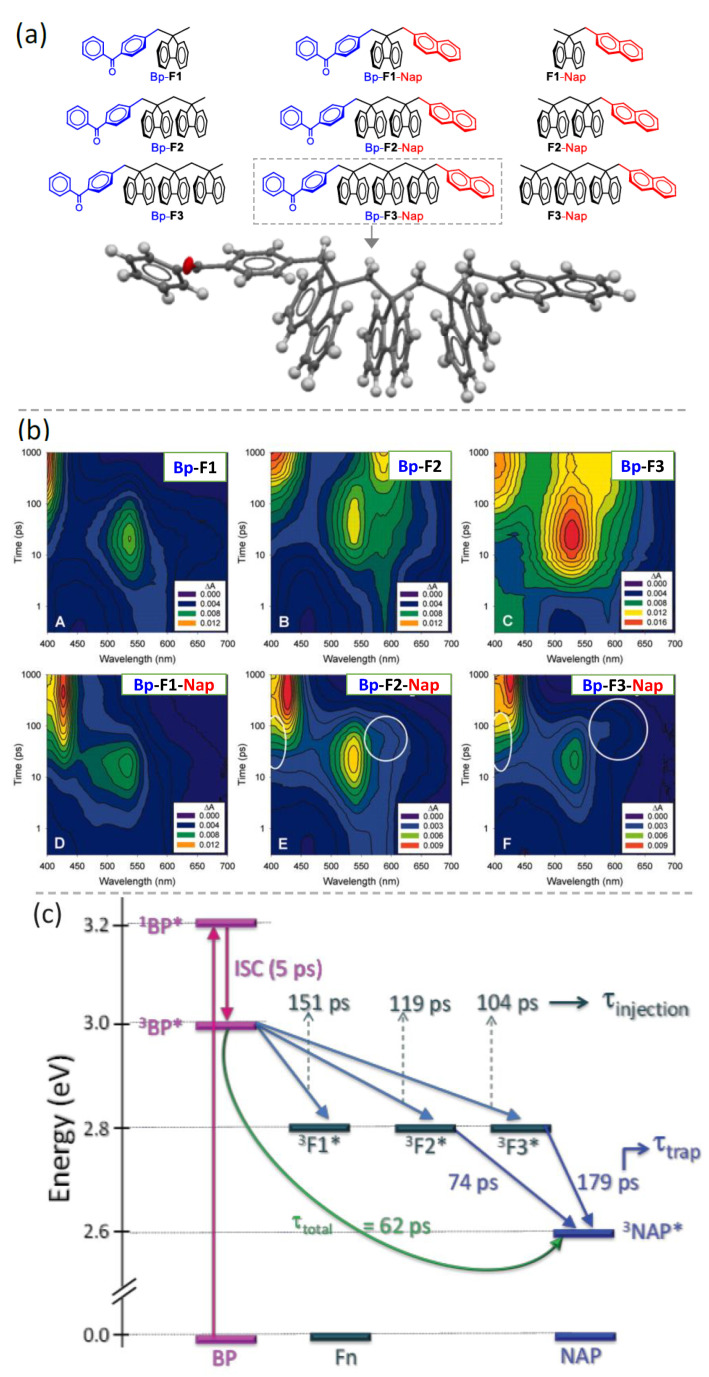
(**a**) Structures of the **Bp**–**Fn**–**Nap** triads, **Bp**–**Fn** and **Fn**–**Nap** diads, and representative X-ray structure of the **Bp**–**F3**–**Nap** triad. (**b**) The 3D transient absorption spectra of **Bp**–**Fn** diads (**A**–**C**) and **Bp**–**Fn**–**Nap** triads (**D**–**F**), where white ovals identify absorption due to the triplet **Fn** bridge-occupied state. (**c**) Energy diagram of **Bp**–**Fn**–**Nap** and **Bp**–**Fn** with observed triplet-energy transfer time constants (Fn* triplet energy of the **Fn**).

The ready availability of polyfluorenes **F2–F6** via versatile synthetic routes, with defined structures and redox and optoelectronic properties, makes them ideal candidates for the study of electron and energy transfer using donor–spacer–acceptor triads, where the **Fn** spacers serve as models for cofacially stacked aromatic π-systems. For example, a series of D–spacer–A molecules—where D is benzophenone (Bp, a triplet energy donor), A is naphthalene (Nap, a triplet energy acceptor), and **F1–F3** are spacers (Figure 18)—and the model compounds were synthesized by adaptation of the strategies outlined in Scheme 6, Scheme 7, Scheme 8, Scheme 9 and Scheme 10. 27.30 Linking of D and A to **Fn** via a single methylene bridge retains their cofacial juxtaposition with the spacer as demonstrated by X-ray crystallography (Figure 18a) [38,45].

Selective photoexcitation of benzophenone moiety at 355 nm in **Bp**–**Fn**–**Nap** molecules was feasible because **Fn** and **Nap** do not absorb at wavelengths longer than 300 nm. The initial nanosecond laser spectroscopic analysis showed that photoexcitation of various **Bp**–**Fn**–**Nap** molecules at 355 nm produces identical transients with the characteristic spectral signature of the naphthalene triplet. These transients are formed within the laser pulse (<10 ns) [30].

Detailed probing of these molecules by femtosecond laser spectroscopy provided complete dynamics of the energy transfer events, beginning from the initial excitation of **Bp** to the trapping of triplet exciton by **Nap** (Figure 18b,c). An examination of the time constants for energy transfer events in model **Bp**–**Fn** diads showed that the exciton is injected into the **Fn** bridge (τ = 151, 119, and 104 ps for n = 1, 2, and 3, respectively), preceded by the rapid interconversion (5 ps) of singlet **Bp** to its triplet. In cases of **Bp**–**Fn**–**Nap** triads, the triplet exciton was injected into the spacer with similar time constants followed by its transfer to **Nap** in cases of **F2** and **F3** spacers (Figure 18b). Surprisingly, however, in the case of the **Bp**–**F1**–**Nap** triad, the transfer of the triplet exciton from **Bp** to **Nap** occurred much faster (62 ps) than the 151 ps observed for the **Bp**–**F1** diad (Figure 18c).

An examination of the complete set of time constants in Figure 18c for various **Bp**–**Fn**–**Nap** triads and **Bp**–**Fn diads** demonstrates that a crossover occurs between single-step tunneling and multi-step hopping of triplet energy transfer as the spacer length increases [38]. Studies are underway to construct similar D–**Fn**–A triads, where D is an electron donor and A is an electron acceptor, for the study of electron transfer dynamics through cofacially arrayed polyfluorene spacers.

## 13. Materials and Methods

### 13.1. Preparation of 9,9-Bis-(2-methyl-allyl)-9H-fluorene (***42***)

Anhydrous THF (25 mL) was added to a Schlenk flask containing fluorene **41** (2.0 g, 12 mmol) under an argon atmosphere and cooled to −40 °C. Dropwise addition of *n*-Butyllithium (5 mL, 12.5 mmol) changed the color to orange, and the reaction was stirred for 10 min. Addition of 3-Chloro-2-methylpropene (1.25 mL, 12.5 mmol) to the reaction mixture, which was then stirred for 30 min, whereupon the orange color disappeared. Aliquots of *n*-Butyllithium (5 mL, 12.5 mmol) and 3-chloro-2-methylpropene (1.25 mL, 12.5 mmol) were added again as described earlier. The work-up was carried out by pouring the mixture into water and extracting the aqueous layer with dichloromethane (2 × 30 mL); the organic extracts were dried over anhydrous magnesium sulfate and evaporated under reduced pressure. The resulting residue was purified by chromatography on silica gel using a 97:3 mixture of hexanes/ethyl acetate as eluent. Yield: 2.60 g (78.8%); mp 60–61 °C (CH_2_Cl_2_/MeOH); ^1^H NMR (CDCl_3_) δ 2.83 (s, 6H), 4.25 (m, 4H), 4.42 (m, 4H), 7.28–7.68 (m, 8H); GC-MS: *m*/*z* = 274 (M^+^) calcd for C_21_H_22_.

### 13.2. Preparation of 1-[9-(2-Oxo-propyl)-9H-fluoren-9-yl]propan-2-one (***43***)

Ozone was bubbled into a solution of **42** (2.5 g, 9.12 mmol) in dry dichloromethane (45 mL) at −78 °C; after 35 min, the reaction mixture turned blue, which indicated that the reaction had reached completion. Next, argon gas was bubbled into the solution until the solution became clear and colorless. Zinc (4.0 g) and glacial acetic acid (10.0 mL) were added at −78 °C and the reaction mixture was stirred for 2 h. Then, it was warmed up to room temperature while stirring for another 2 h and then filtered through a short layer of celite. The filtrate was poured into water (30 mL) and extracted with ethyl ether (3 × 40 mL). The combined organic extracts were washed with water, dried over anhydrous magnesium sulfate and evaporated under reduced pressure. The resulting residue was purified by chromatography on silica gel using a 94:6 mixture of hexanes/ethyl acetate as eluent. Yield: 0.9 g (36%); mp 65–67 °C (Ether_/_MeOH); ^1^H NMR (CDCl_3_) δ 1.94 (s, 6H), 3.25 (m, 4H), 7.25–7.75 (m, 8H); ^13^C NMR (CDCl_3_) δ 31.98, 50.87, 120.06, 124.54, 127.6, 127.76, 139.44, 149.61, 206.78; GC-MS: *m*/*z* = 278 (M^+^) calcd for C_19_H_18_O_2_.

### 13.3. Preparation of 2-Biphenyl-2-yl-1-[9-(2-biphenyl-2-yl-2-hydroxy-propyl)-9H-fluoren-9-yl]propan-2-ol (***44***)

A solution of 2-biphenylmagnesium bromide was prepared from 2-bromobiphenyl (0.92 g, 3.96 mmol) and excess magnesium turnings (0.23 g, 8 mmol) in anhydrous tetrahydrofuran (25 mL) under an argon atmosphere by refluxing for 4 h. The Grignard solution thus obtained was transferred after cooling to room temperature to another Schlenk flask containing **43** (0.5 g, 1.79 mmol). The mixture was refluxed overnight, cooled to room temperature, and quenched with dilute hydrochloric acid (5%, 10 mL). The organic layer was separated and the aqueous phase was extracted with dichloromethane (3 × 15 mL). The combined organic extracts were dried over anhydrous magnesium sulfate and filtered. After evaporation of the solvent under vacuum, the resulting residue was purified by chromatography on silica gel using a 97:3 mixture of hexanes/ethyl acetate as eluent to recover biphenyl, the starting material.

### 13.4. Preparation of 9-Methyl-9H-fluorene (***45***)

Anhydrous THF (50 mL) was added to a Schlenk flask containing fluorene (5.0 g, 30.12 mmol) under an argon atmosphere and cooled to −40 °C. Dropwise addition of *n*-Butyllithium (13.2 mL, 33 mmol) was carried out, during which the color changed to orange. The reaction was stirred for 10 min and then iodomethane (2.5 mL, 40 mmol) was added. The mixture was stirred for 15 min during which the color disappeared. The work-up was carried out by pouring the reaction mixture into water (50 mL) followed by extraction with dichloromethane (3 × 30 mL). The combined organic extracts were dried over anhydrous magnesium sulfate and evaporated under reduced pressure. Yield: 4.9 g (90%); mp 61–63 °C (CH_2_Cl_2_/MeOH) (mp 62–64 °C); ^1^H NMR (CDCl_3_) δ 1.52 (d, 3H), 3.97 (q, 1H), 7.12–7.80 (m, 8H); ^13^C NMR (CDCl_3_) δ 18.98, 42.91, 119.63, 123.76, 126.62, 140.10, 148.47; GC-MS: *m*/*z* = 180 (M^+^) calcd for C_14_H_12_.

### 13.5. Preparation of 2,3-Bis(9-methylfluorene)methylpropene (***46***)

Anhydrous THF (50 mL) was added to a Schlenk flask containing **45** (5.0 g, 27.8 mmol) under an argon atmosphere and cooled to −30 °C. A quantity of *n*-Butyllithium (11.5 mL, 28.5 mmol) was added dropwise, during which the color changed to orange. The reaction was stirred for 10 min, and 3-chloro-2-chloromethylpropene (1.7 mL, 20 mmol) was added. The reaction mixture was stirred for 1 h and the color disappeared. The work-up was carried out by pouring the reaction mixture into water followed by extraction with dichloromethane (3 × 20 mL). The combined organic layers were dried over anhydrous magnesium sulfate and evaporated under reduced pressure. Yield: 5.6 g (97%); mp 85–87 °C (CH_2_Cl_2/_MeOH); ^1^H NMR (CDCl_3_) δ 1.28 (s, 6H), 2.11 (s, 4H), 6.28 (m, 1H), 3.75 (s, 2H), 7.16–7.63 (m, 16H); ^13^C NMR (CDCl_3_) δ 27.99, 46.92, 51.68, 116.81, 119.94, 123.63, 126.79, 126.93, 140.03, 141.09, 151.29; GC-MS: *m*/*z* = 412 (M^+^) calcd for C_32_H_28_.

### 13.6. Preparation of 1,3-Bis(9-methyl-9H-fluoren-9-yl)propan-2-one (***47***)

Ozone was bubbled into a solution of **46** (5.50 g, 13.35 mmol) in dry dichloromethane (55 mL) at −78 °C. After 2 h, the reaction mixture turned blue, which indicated that the reaction had reached completion, and then argon gas was bubbled through it until it became clear and colorless. Zinc powder (5.8 g) and glacial acetic acid (14 mL) were added, and the mixture was warmed up to room temperature and stirred overnight. The reaction mixture was filtered through a short layer of celite. The filtrate was poured into water (50 mL), and the aqueous layer was extracted with ethyl ether (3 × 40 mL). The combined organic extracts were washed with water, dried over anhydrous magnesium sulfate, and evaporated under reduced pressure. Yield: 4.1g (77.8%); mp 96–98 °C (CH_2_Cl_2/_MeOH); ^1^H NMR (CDCl_3_) δ 1.31 (s, 6H), 2.53 (s, 4H), 7.23–7.68 (m, 16H); ^13^C NMR (CDCl_3_) δ 26.16, 49.22, 52.92, 119.77, 123.19, 126.98, 127.04, 139.13, 150.35, 204.61.

### 13.7. Preparation of 2-Biphenyl-2-yl-1,3-bis(9-methyl-9-H-fluoren-9-yl)propan-2-ol (***48***)

(A) A solution of 2-biphenyl magnesium bromide was prepared from 2-bromobiphenyl (1.5 g, 6.2 mmol) and excess magnesium turnings (0.45 g, 18.6 mmol) in anhydrous tetrahydrofuran (40 mL) under an argon atmosphere and refluxing for 4 h. The Grignard solution thus obtained was transferred at room temperature to another Schlenk flask containing **47** (2.0 g, 4.83 mmol). The mixture was refluxed overnight, cooled to room temperature, and quenched with dilute hydrochloric acid (5%, 10 mL). The organic layer was separated, and the aqueous phase was extracted with dichloromethane (3 × 20 mL). The combined organic extracts were dried over magnesium sulfate and filtered. After evaporation of the solvent under reduced pressure, the resulting residue was purified by chromatography on silica gel using a 97:3 mixture of hexanes/ethyl acetate as eluent to recover the starting material and biphenyl.

(B) 2-Biphenyl lithium was prepared according to a published procedure [39]. A quantity of *tert*-butyllithium (3.13 mL, 4.84 mmol, 1.7 M in pentane) was added to a solution of 2-iodobiphenyl (0.677 g, 2.42 mmol) in ether (10 mL) at −78 °C over 30 min. The resulting slurry was stirred at −78 °C for 1 h, then **47** (1.0 g, 2.42 mmol) dissolved in ether (10 mL) was added dropwise over 10 min. The solution was warmed to room temperature for over 30 min and poured into water. The aqueous layer was extracted with ether (3 × 15 mL), and the combined organic layers were washed with brine and dried over magnesium sulfate. After evaporation of the solvent under reduced pressure, the resulting residue was purified by chromatography on silica gel using a 97:3 mixture of hexanes/ethyl acetate as eluent to recover the starting material and biphenyl.

### 13.8. Preparation of 9-[(Fluoren-9-yl)-methyl]flourene (***55***)

Fluorene (1.66 g, 10 mmol) was placed in a 100 mL Schlenk flask under an argon atmosphere. After addition of DMF (12.5 mL) and potassium *tert*-butoxide (1.12 g, 10 mmol), the mixture turned red. Then, 9-Methanol-fluorene (1.96 g, 10 mmol) was added to the reaction mixture and stirred for 15 min at room temperature. The mixture was poured onto water (20 mL) and then filtered to provide **55**. Yield: 2.9 g (86%); mp 210–212 °C (CH_2_Cl_2/_MeOH) (mp 212–213 °C); ^1^H NMR (CDCl_3_) δ 2.23 (t, *J* = 7.6 Hz, 2H), 4.45 (t, *J* = 7.6 Hz, 2H), 7.25 (t, *J* = 7.4 Hz, 4H), 7.39 (t, *J* = 7.4 Hz, 4H), 7.54 (d, *J* = 7.5 Hz, 4H), 7.81 (d, *J* = 7.5 Hz, 4H); ^13^C NMR (CDCl_3_) δ 39.37, 46.31, 119.87, 124.75, 126.65, 126.98, 140.53, 146.95; GC-MS: *m*/*z* = 344 (M^+^) calcd for C_27_H_20_.

### 13.9. Preparation of 9,9-Bis((9H-fluoren-9-yl)methyl)-9H-fluorene (***63***)

A sample of **55** (1.73 g, 5 mmol) was placed in a 100 mL Schlenk flask under an argon atmosphere. After addition of DMF (12.5 mL) and potassium *tert*-butoxide (0.56 g, 5 mmol) the mixture was stirred for 5 min at room temperature, then 9-methanol-flourene (0.98 g, 5 mmol) was added to the reaction mixture and it was stirred for another 1h. The mixture was worked up by quenching it with dilute hydrochloric acid (5%, 30 mL) and then filtered. The crude product was purified by column chromatography on silica gel using a 90:10 mixture of hexanes/ethyl acetate as eluent to produce **66.** Yield: 2.5 g (72%); mp 274–276 °C; ^1^H NMR (CDCl_3_) δ 2.80 (d, *J* = 4.5 Hz, 4H), 3.25 (t, *J* = 4.7 Hz, 2H), 6.62 (d, *J* = 7.1 Hz, 4H), 7.04 (t, *J* = 4.7 Hz, 4H), 7.22 (t, *J* = 4.7 Hz, 4H), 7.48–7.57 (m, 8 H), 7.72–7.5 (m, 2H), 7.86–7.88 (m, 2H); ^13^C NMR (CDCl_3_) δ: 44.74, 46.02, 55.70, 119.37, 120.93, 124.99, 125.50, 126.70, 126.76, 127.58, 128.26, 140.53, 141.95, 148.58, 148.90.

### 13.10. Preparation of Bis(9-((9H-fluoren-9-yl)methyl)-9H-fluoren-9-yl)methane (***64***)

A sample of **55** (1.73 g, 5 mmol) was placed in a 100 mL Schlenk flask under an argon atmosphere. After addition of DMF (12.5 mL) and potassium *tert*-butoxide (0.56 g, 5 mmol), the mixture was stirred for 5 min at room temperature. Then, 9-methanol-flourene (1.96 g, 10 mmol) was added to the reaction mixture and it was stirred for another 1h. The mixture was worked up by quenching it with dilute hydrochloric acid (5%, 50mL), and then filtered. The crude product was purified by column chromatography on silica gel using a 90:10 mixture of hexanes/ethyl acetate as eluent to produce **64**. Yield: 2.5 g (72%); mp 253–255 °C (CH_2_Cl_2_/MeOH); ^1^H NMR (CDCl_3_) δ 2.59 (d, *J* = 4.6 Hz, 4H), 2.80 (t, *J* = 4.4 Hz, 2H), 3.30 (s, 2H), 6.39 (d, *J* = 7.6 Hz, 4H), 6.93–7.22 (m, 32 H), 7.49 (d, *J* = 7.6 Hz, 4H); ^13^C NMR (CDCl_3_) δ 43.77, 47.76, 49.78, 54.03, 61.41, 119.178, 119.66, 124.95, 124.99, 126.16, 126.52, 126.64, 126.71, 140.36. 141.37, 147.65, 148.88.

### 13.11. Preparation of 9-Methyl-9H-fluorene-d9 (***70***)

Anhydrous THF (20 mL) was added to a Schlenk flask containing fluorene-*d_8_* (0.49 g, 2.78 mmol) under an argon atmosphere and cooled to −30 °C. A quantity of *n*-Butyllithium (1.12 mL, 3.1 mmol) was added dropwise, with the color changing to orange during the addition. The reaction was stirred for 10 min and then iodomethane (0.5 mL, 8 mmol) was added. The mixture was stirred for 15 min, during which the color disappeared. The work-up was carried out by pouring the reaction mixture into dilute hydrochloric acid (5%, 20 mL) followed by extraction with dichloromethane (3 × 20 mL). The combined organic extracts were dried over anhydrous magnesium sulfate and evaporated under reduced pressure. Yield: 0.41g (78%); mp 61–63 °C (CH_2_Cl_2_/MeOH); ^1^H NMR (CDCl_3_) δ 1.58 (s, 3H); ^13^C NMR (CDCl_3_) δ 18.43, 42.86, 140.10, 148.47; GC-MS: *m*/*z* = 189 (M^+^) calcd for C_14_H_3_D_9_.

### 13.12. Preparation of 9-(2-Bromoallyl)-9-methyl-9H-fluorene-d8 (***71***)

To a suspension solution of **70** (0.41 g, 2.17 mmol) in toluene (15 mL) under an argon atmosphere at room temperature, a sodium hydroxide 50% solution (15 mL) was added, then 2,3-dibromopropene (0.4 mL, 4 mmol) was added to the reaction mixture. Tetra-n-butylammonium bromide (80 mg) was added as a phase-transfer catalyst. The reaction mixture was stirred overnight at room temperature. The work-up was performed by adding ethyl acetate (10 mL) and separating the organic layer. The aqueous layer was washed with ethyl acetate (2 × 10 mL). The combined organic extracts were washed with dilute hydrochloric acid (5%, 15 mL) and water, dried over magnesium sulfate, and evaporated under reduced pressure. The oily crude product was purified by column chromatography on silica gel using a 98:2 mixture of hexanes/ethyl acetate as eluent to yield **71**. Yield: 0.16 g (30%); (CH_2_Cl_2/_MeOH); ^1^H NMR (CDCl_3_) δ 1.58 (s, 3H), 3.18 (s, 2H), 4.94 (d, 1H), 5.17 (d, 1H,); ^13^C NMR (CDCl_3_) δ 27.27, 50.68, 51.01, 120.39, 128.63, 140.21, 148.42; GC-MS: *m*/*z* = 306 (M^+^) calcd for C_17_H_7_D_8_Br.

### 13.13. Preparation of 9-(2-Biphenyl-2-yl-allyl)-9-methyl-9H-fluorene-d8 (***72***)

A solution of 2-biphenylmagnesium bromide was prepared from 2-bromobiphenyl (0.25 g, 10 mmol) and excess magnesium turnings (0.30 g, 12.5 mmol) in anhydrous THF (20 mL), and 5 drops of dibromoethane were added to activate the reaction under an argon atmosphere and refluxing for 4 h. The Grignard solution thus obtained was transferred at room temperature to another Schlenk flask containing **42** (0.16 g, 0.50 mmol), and a catalytic amount of *bis*(triphenylphosphine)palladium dichloride (60 mg) was added. The resulting yellow mixture was refluxed overnight, cooled to room temperature, and quenched with dilute hydrochloric acid (5%, 10 mL). The organic layer was separated and the aqueous phase was extracted with dichloromethane (3 × 15 mL). The combined organic extracts were dried over magnesium sulfate and filtered. Evaporation of the solvent under reduced pressure yielded a brown residue that was filtered through a short pad of silica gel with hexanes as eluent, after which the resulting product was further purified by column chromatography on silica gel using a 95:5 mixture of hexanes/ethyl acetate as eluent. Yield: 0.15 g (76%); mp 127–129 °C (CH_2_Cl_2_/MeOH); ^1^H NMR (CDCl_3_) δ 1.07 (s, 3H), 2.40 (2H, s), 4.42 (d, 1H), 4.66 (d, 1H), 6.01 (d, 1H) 6.64–7.7.34 (m, 8H); ^13^C NMR (CDCl_3_) δ 27.52, 45.59, 51.33, 119.25, 126.76, 126.82, 126.90, 128.27, 129.13, 129.15, 129.29, 130.01, 138.05, 140.15, 142.22, 147.54, 148.51.

### 13.14. Preparation of Bis(9-methyl-9H-fluoren-9-yl)methane-d8 (***F2*-d8**)

To a cold (0 °C) solution of **72** (75 mg, 0.2 mmol) in dry dichloromethane (10 mL), a few drops (5) of methanesulfonic acid were added, and the reaction mixture was allowed to warm up to room temperature and stirred for 6 h. The work-up was performed by adding saturated solution of sodium bicarbonate (10 mL) to remove the excess acid, followed by extraction with dichloromethane (3 × 15 mL). The combined organic extracts were dried over magnesium sulfate and evaporated under reduced pressure, and the resulting product was further purified by column chromatography on silica gel using a 98:2 mixture of hexanes/ethyl acetate as eluent. Yield: 55 mg (74%); mp 175–177 °C (CH_2_Cl_2/_MeOH); ^1^H NMR (CDCl_3_) δ 1.23 (s, 3H), 3.07 (s, 2H), 6.83–7.12 (m, 8H); ^13^C NMR (CDCl_3_) δ 29.93, 49.35, 49.92, 118.61, 119.14, 123.37, 125.92, 136.10, 150.68.

### 13.15. Preparation of Di(9H-fluoren-9-yl)methane-d_16_ (***73***)

Fluorene-*d_10_* (1.0 g, 5.7 mmol) was placed in a 100 mL Schlenk flask under an argon atmosphere. After addition of DMF (10 mL) and potassium *tert*-butoxide (0.64 g, 5.7 mmol), the mixture turned red. Paraformaldehyde (85 mg 2.8 mmol) was added to the reaction mixture, and it was stirred for 1 h at room temperature before it was poured into dilute hydrochloric acid (5%, 20 mL) and then filtered. Yield: 1.01 g (98%); mp 210–212 °C (CH_2_Cl_2/_MeOH); ^1^H NMR (CDCl_3_) δ 2.23 (t, 2H), 4.45 (t, 2H); GC-MS: *m*/*z* = 361 (M^+^) calcd for C_27_H_4_D_16_.

### 13.16. Preparation of 9,9-Bis-(2-bromo-allyl)-9H-fluorene-d8 (***74***)

To a suspension solution of fluorene-*d_8_* (0.5 g, 2.85 mmol) in toluene (15 mL) under an argon atmosphere at room temperature, sodium hydroxide 50% solution (15 mL) was added, then 2,3-dibromopropene (1.0 mL, 8.55 mmol) was added to the reaction mixture. Tetra-butyl ammonium bromide (100 mg) was added as a phase-transfer catalyst. The reaction mixture was stirred overnight at room temperature. The work-up was carried out by adding ethyl acetate (15 mL) and separating the organic layer. The aqueous layer was washed with ethyl acetate (2 × 15 mL). The combined organic extracts were washed with dilute hydrochloric acid (5%, 15 mL) and water, dried over magnesium sulfate, and evaporated under reduced pressure. The oily crude product was purified by column chromatography on silica gel using a 98:2 mixture of hexanes/ethyl acetate as eluent to produce **74**. Yield: 0.53 g (44%); ^1^H NMR (CDCl_3_) δ 3.24 (s, 2H), 4.89 (d, 1H), 5.12 (d, 1H,); ^13^C NMR (CDCl_3_) δ 49.95, 54.89, 120.99, 126.88, 141.03, 148.53.

### 13.17. Preparation of 9,9-Bis(2-biphenyl-2-yl-allyl)-9H-fluorene-d8 (***75***)

A solution of 2-biphenylmagnesium bromide was prepared from 2-bromobiphenyl (0.75 g, 3.21 mmol) and excess magnesium turnings (0.38 g, 15.6 mmol) in anhydrous THF (25 mL), and 5 drops of dibromoethane were added to activate the reaction under an argon atmosphere and refluxing for 4 h. The Grignard solution thus obtained was transferred at room temperature to another Schlenk flask containing **74** (0.53 g, 1.29 mmol), and a catalytic amount of *bis*(triphenylphosphine)palladium dichloride (0.1 g) was added. The resulting yellow mixture was refluxed overnight, cooled to room temperature, and quenched with dilute hydrochloric acid (5%, 30 mL). The organic layer was separated and the aqueous phase was extracted with dichloromethane (3 × 50 mL). The combined organic extracts were dried over magnesium sulfate and filtered. Evaporation of the solvent under reduced pressure yielded a brown residue that was filtered through a short pad of silica gel with hexanes as eluent, after which the resulting product was further purified by column chromatography on silica gel using a 95:5 mixture of hexanes/ethyl acetate as eluent. Yield: 0.56 g (79%); mp 151–153 °C (CH_2_Cl_2_/MeOH); ^1^H NMR (CDCl_3_) δ 2.07 (s, 4H), 4.23 (d, 2H), 4.51 (d, 2H), 5.93 (d, 2H) 6.65–7.47 (m, 16H); ^13^C NMR (CDCl_3_) δ 45.51, 55.77, 119.51, 126.57, 126.79, 128.16, 128.99, 129.98, 137.81, 140.12, 141.07, 141.98, 142.14, 146.79, 148.31, 148.63.

### 13.18. Preparation of 9,9-Bis((9-methyl-9H-fluoren-9-yl)methyl)-9H-fluorene-d_8_ (***F3*-d8**)

To a cold (0 °C) solution of **75** (0.56 g, 1 mmol) in dry dichloromethane (20 mL), a few drops (10) of methanesulfonic acid were added, and the reaction mixture was warmed up to room temperature and stirred for 4 h. The work-up was carried out by adding a saturated solution of sodium bicarbonate (30 mL) to remove the excess acid followed by extraction with dichloromethane (2 × 20 mL). The combined organic extracts were washed with water, dried over magnesium sulfate, and evaporated under reduced pressure. The resulting product was further purified by column chromatography on silica gel using a 95:5 mixture of hexanes/ethyl acetate as eluent. Yield: 0.22 g (40%); mp 227–229 °C (CH_2_Cl_2/_MeOH); ^1^H NMR (CDCl_3_) δ 1.10 (s, 6H), 2.90 (s, 4H), 6.62–7.02 (m, 16H); ^13^C NMR (CDCl_3_) δ 30.23, 49.46, 50.88, 52.96, 118.93, 123.19, 125.67, 125.75, 139.48, 150.25.

### 13.19. Preparation of 9-(2-Bromo-allyl)-9-[(9-(2-bromo-allyl)-fluoren-9-yl)methyl]fluorene-d16 (***76***)

To a suspension solution of **73** (1.01 g, 2.9 mmol) in toluene (15 mL) under an argon atmosphere at room temperature, sodium hydroxide 50% solution (15 mL) was added, and then 2,3-dibromopropene (1.0 mL, 8.55 mmol) was added to the reaction mixture. Tetra-butyl ammonium bromide (100 mg) was added as a phase-transfer catalyst. The reaction mixture was stirred overnight at room temperature. The work-up was performed by adding ethyl acetate (15 mL) and separating the organic layer. The aqueous layer was washed with ethyl acetate (2 × 15 mL). The combined organic extracts were washed with dilute hydrochloric acid (5%, 15 mL) and water, dried over magnesium sulfate, and evaporated under reduced pressure. The crude product was purified by column chromatography on silica gel using a 95:5 mixture of hexanes/ethyl acetate as eluent. Yield: 0.51 g (35%); mp 172–175 °C (CH_2_Cl_2/_MeOH); ^1^H NMR (CDCl_3_) δ 3.01 (s, 2H), 3.26 (s, 2H), 4.59 (d, 2H), 4.96 (d, 2H).

### 13.20. Preparation of 9-(2-Biphenyl-2-yl-allyl)-9-[(9-(2-biphenyl-2-yl-allyl)fluoren-9-yl)methyl]fluorene-d16 (***77***)

A solution of 2-biphenylmagnesium bromide was prepared from 2-bromobiphenyl (0.4 g, 1.7 mmol) and excess magnesium turnings (0.2 g, 8.5 mmol) in anhydrous THF (20 mL), and 5 drops of dibromoethane were added to activate the reaction under an argon atmosphere and refluxing for 4 h. The Grignard solution thus obtained was transferred at room temperature to another Schlenk flask containing **76** (0.51 g, 0.85 mmol), and a catalytic amount of *bis*(triphenylphosphine) palladium dichloride (0.1 g) was added. The resulting yellow mixture was refluxed overnight, cooled to room temperature, and quenched with dilute hydrochloric acid (5%, 10 mL). The organic layer was separated and the aqueous phase was extracted with dichloromethane (3 × 15 mL). The combined organic extracts were dried over magnesium sulfate and evaporated under reduced pressure to produce a brown residue that was filtered through a short pad of silica gel with hexanes as eluent. The resulting product was further purified by column chromatography on silica gel using a 95:5 mixture of hexanes/ethyl acetate as eluent. Yield: 0.48 g (76%), mp 146–148 °C (CH_2_Cl_2/_MeOH); ^1^H NMR (CDCl_3_) δ 2.13 (s, 4H), 2.33 (s, 2H), 4.12 (d, 2H), 4.51 (d, 2H), 5.76 (2H, d), 6.98–7.43 (m, 16H); ^13^C NMR (CDCl_3_) δ 47.29, 48.18, 53.94, 119.75, 126.43, 126.73, 126.82, 128.25, 128.98, 129.14, 129.90, 137.69, 140.56, 142.95, 142.45, 146.25, 147.16.

### 13.21. Preparation of Bis(9-((9-methyl-9H-fluoren-9-yl)methyl)-9H-fluoren-9-yl)methane-d16 (***F4*-d16**)

To a cold (0 °C) solution of **77** (0.48 g, 0.67 mmol) in dry dichloromethane (25 mL), a few drops (5) of methanesulfonic acid were added, and the reaction mixture was allowed to warm up to room temperature and stirred for 4 h. The work-up was carried out by adding a saturated solution of sodium bicarbonate (30 mL) to remove the excess acid followed by extraction with dichloromethane (3 × 15 mL). The combined organic extracts were dried over magnesium sulfate and evaporated under reduced pressure. Yield: 0.15 g (36%); mp 215–217 °C (CH_2_Cl_2/_MeOH); ^1^H NMR (CDCl_3_) δ 1.04 (s, 6H), 2.71 (s, 6H), 6.09–6.99 (m, 16H); ^13^C NMR (CDCl_3_) δ 30.28, 49.35, 50.87, 52.90, 118.88, 123.09, 124.21, 124.26, 125.58, 125.69, 139.38, 140.38, 146.40, 150.21.

## 14. Conclusions

Syntheses of cofacially arrayed multi-layered π-systems based on traditional cyclophanes are scarce and not readily amenable to the structural modifications required for construction of the D–spacer–A triads owing to the synthetic difficulties associated with the preparation of sandwich-like structures. By controlling the number of C–C bond rotors and utilizing a larger aromatic π-system in a 1,3-diarylalkane platform, we developed versatile synthetic strategies for the preparation of monodisperse cofacially stacked polyfluorenes. These synthetic strategies also allow the ready incorporation of polyfluorenes into D–spacer–A triads by linking donor and acceptor groups to the polyfluorene spacers via single methylenes. A combination of ^1^H NMR spectroscopy, X-ray crystallography, electrochemistry, and photoelectron spectroscopy have demonstrated the cofacial stacking of multiple fluorenes in **F2–F6** and hole and electron delocalization amongst the stacked fluorenes. We also demonstrated, using D–spacer–A triads, that triplet energy transfer through π-stacked polyfluorenes occurs via tunneling and hopping mechanisms depending on the number of fluorenes in the polyfluorene spacer.

We believe that the ready availability of rigidly held π-stacked polyfluorenes, described herein, with well-defined redox and optoelectronic properties provides an ideal scaffold for the study of electron and energy transfer in D–spacer–A triads where the **Fn** spacers will serve as models for cofacially stacked π-systems.

## Figures and Tables

**Table 1:** The *E*_ox1_ of cyclophane-like bichromophoric molecules with varying interplanar angles (obtained from the X-ray structures) and their corresponding model compounds [12].

Monomer	*E*ox (V) ^a^	Cyclophane		*E*ox (V) ^a^	D (V)
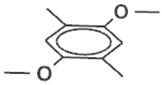 **23**	0.66	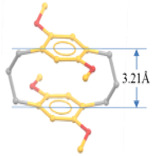	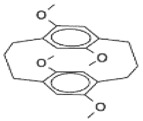	0.33	0.33
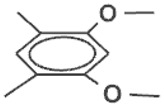 **24**	0.73	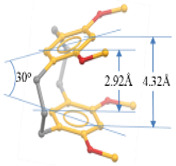	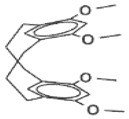	0.35	0.38
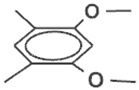 **26**	0.77	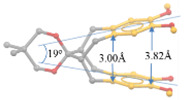	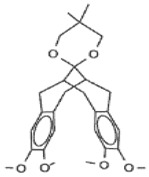	0.42	0.35
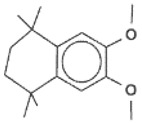 **27**	0.82	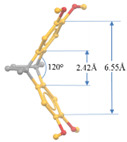	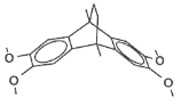	0.57	0.25

^a^ The *E*ox vs. Fc/Fc^+^ were measured in CH_2_Cl_2_ containing (*n*-Bu)_4_NPF_6_ at *v* = 200 mV/sec at 22 °C. (*n*-Bu)_4_NPF_6_ is tetrabutylammonium hexafluorophosphate.

## Data Availability

The authors confirm that the data supporting the findings of this study are available within the article references.
